# Modelling of the tsunami from the December 22, 2018 lateral collapse of Anak Krakatau volcano in the Sunda Straits, Indonesia

**DOI:** 10.1038/s41598-019-48327-6

**Published:** 2019-08-16

**Authors:** Stephan T. Grilli, David R. Tappin, Steven Carey, Sebastian F. L. Watt, Steve N. Ward, Annette R. Grilli, Samantha L. Engwell, Cheng Zhang, James T. Kirby, Lauren Schambach, Muslim Muin

**Affiliations:** 1 Department of Ocean Engineering, University of Rhode Island (URI), Narragansett, RI USA; 2 Graduate School of Oceanography, University of Rhode Island (URI), Narragansett, RI USA; 3 British Geological Survey (BGS), Nottingham, UK; 4 University College London (UCL), London, UK; 5 School of Geography, Earth and Environmental Sciences, University of Birmingham (UB), Birmingham, UK; 6 University of California Santa Cruz (UCSC), Santa Cruz, CA USA; 7 Center for Applied Coastal Research, University of Delaware (UD), Newark, DE USA; 8 Bandung Institute of Technology, Bandung, Indonesia

**Keywords:** Natural hazards, Ocean sciences, Planetary science

## Abstract

On Dec. 22, 2018, at approximately 20:55–57 local time, Anak Krakatau volcano, located in the Sunda Straits of Indonesia, experienced a major lateral collapse during a period of eruptive activity that began in June. The collapse discharged volcaniclastic material into the 250 m deep caldera southwest of the volcano, which generated a tsunami with runups of up to 13 m on the adjacent coasts of Sumatra and Java. The tsunami caused at least 437 fatalities, the greatest number from a volcanically-induced tsunami since the catastrophic explosive eruption of Krakatau in 1883 and the sector collapse of Ritter Island in 1888. For the first time in over 100 years, the 2018 Anak Krakatau event provides an opportunity to study a major volcanically-generated tsunami that caused widespread loss of life and significant damage. Here, we present numerical simulations of the tsunami, with state-of the-art numerical models, based on a combined landslide-source and bathymetric dataset. We constrain the geometry and magnitude of the landslide source through analyses of pre- and post-event satellite images and aerial photography, which demonstrate that the primary landslide scar bisected the Anak Krakatau volcano, cutting behind the central vent and removing 50% of its subaerial extent. Estimated submarine collapse geometries result in a primary landslide volume range of 0.22–0.30 km^3^, which is used to initialize a tsunami generation and propagation model with two different landslide rheologies (granular and fluid). Observations of a single tsunami, with no subsequent waves, are consistent with our interpretation of landslide failure in a rapid, single phase of movement rather than a more piecemeal process, generating a tsunami which reached nearby coastlines within ~30 minutes. Both modelled rheologies successfully reproduce observed tsunami characteristics from post-event field survey results, tide gauge records, and eyewitness reports, suggesting our estimated landslide volume range is appropriate. This event highlights the significant hazard posed by relatively small-scale lateral volcanic collapses, which can occur *en-masse*, without any precursory signals, and are an efficient and unpredictable tsunami source. Our successful simulations demonstrate that current numerical models can accurately forecast tsunami hazards from these events. In cases such as Anak Krakatau’s, the absence of precursory warning signals together with the short travel time following tsunami initiation present a major challenge for mitigating tsunami coastal impact.

## Introduction

In the evening of December 22, 2018, a lateral collapse on the southwest flank of Anak Krakatau (AK) volcano, Indonesia, generated a tsunami along adjacent coastlines in which at least 437 people died^1^. This was the most damaging volcanically-generated tsunami since the 1883 eruption of Krakatau, which killed 36,000 people^2,3^, and the lateral-collapse generated tsunami at Ritter Island, Papua New Guinea, in 1888^4^. Over the past 20 years, catastrophic tsunamis in Papua New Guinea (1998), the Indian Ocean (2004), and Japan (2011) have driven major advances in understanding of earthquakes and submarine landslides as tsunami sources, from developments in constraining source mechanisms, their geographical distribution, and tsunami numerical modelling capability. Tsunamis from volcanic sources, including both eruptions and landslides, have resulted in significant losses of life and property^5,6^, accounting for 20% of all volcanic fatalities over the past 400 years^7^, but there are no direct observations of a large-scale volcanic tsunami since that at Ritter Island^8^. Tsunamis from lateral collapses have been widely modelled, including very large landslide scenarios on ocean islands e.g.,^9–11^, as well as smaller scale landslides more comparable to the AK collapse^12–14^. However, because of the paucity of historical examples the results of these models are not fully validated, and aspects of both landslide source mechanisms e.g.,^15–17^ and tsunami behaviour e.g.,^10,11^ remain poorly understood and challenging to model.

**Figure 1:**
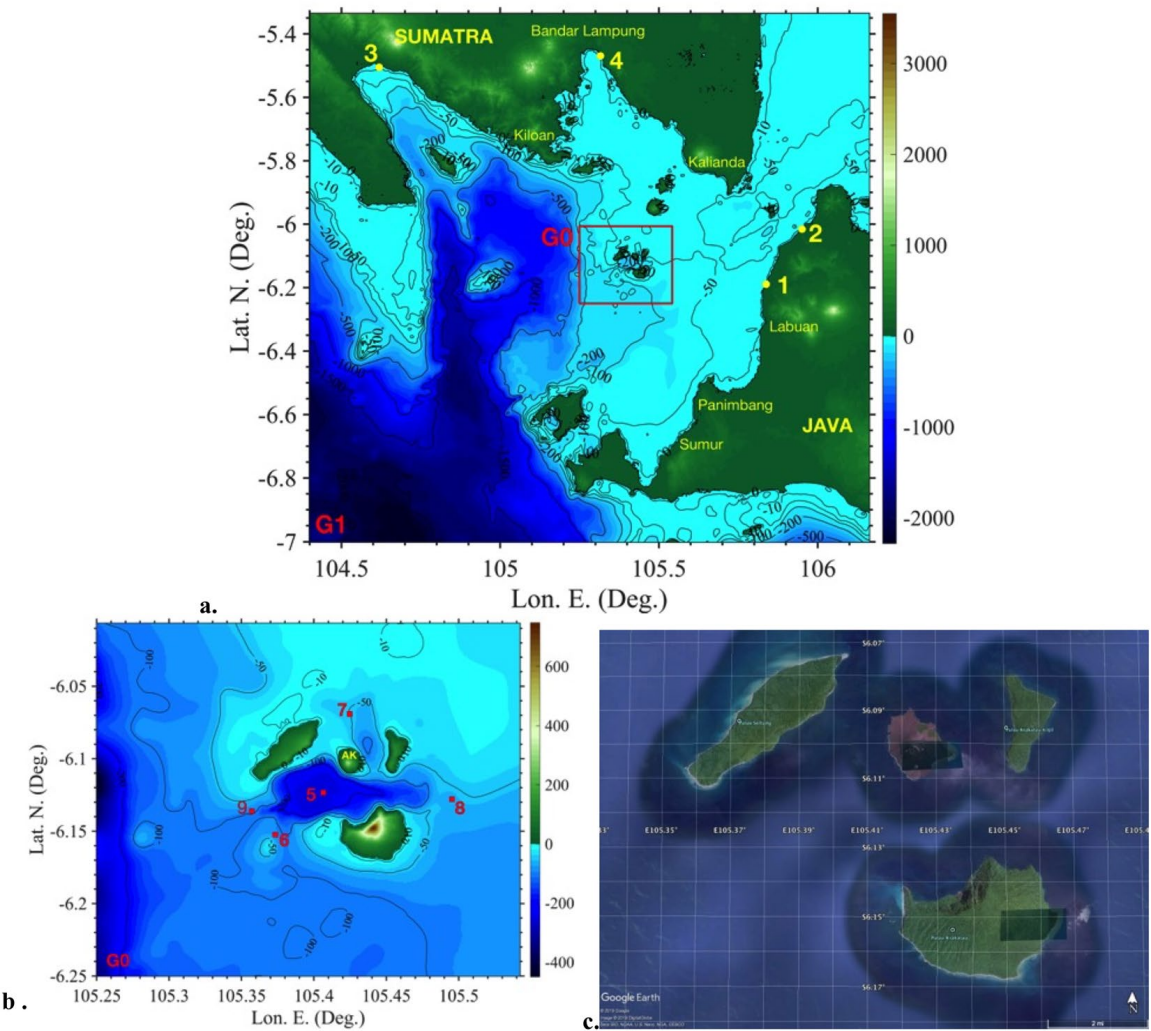
Sunda Straits area, for AK collapse and tsunami simulations. (**a**,**b**) Bathymetry/topography (colour scales and contours in meter; Mean Sea Level bathymetry/topography dataset obtained from^12^, +1.5 m for tide), tide (1–4) and wave gauges (5–9) (Table 1): (**a)** FUNWAVE G1 (100 m horizontal resolution grid); (**b**) NHWAVE G0 grid (90 m horizontal resolution and 5 vertical *σ*-layers), with footprint marked by red box in (**a**). (**c**) Pre-collapse image showing AK and Sertung (W), Panjang/Kitjil (E), and Rakata/Krakatau. Maps in (**a**,**b)** were produced by the authors based on the bathymetry/topography dataset, using MATLAB version 2017b (**c**), map data is from: GOOGLE EARTH, 2019 DigitalGlobe (Data: SIO, NOAA, U.S. Navy, NGA, GEBCO).

Anak Krakatau (Fig. 1), a small composite volcanic cone that developed within the 250 m deep caldera of Krakatau, emerged subaerially in 1928^18–20^. It is situated on the northeast margin of the caldera wall and is aligned with the feeder vents of the 1883 Krakatau eruption^2^. During the past 90 years, it has grown from a submarine volcano to a subaerial edifice with a pre-collapse height of about 335 m. Retreats of the coastline to the SW by several hundred metres in 1934, 1935 and 1950^20^ align with the 2018 collapse and imply instability of the edifice on the SW side, as a result of its position on the submerged scarp of the 1883 caldera. Early activity at Anak Krakatau was dominated by phreatomagmatic explosions. The first lava flows were erupted between 1960 and 1963 as the vent site became fully subaerial^21^ and numerous subsequent eruptions have mostly featured expansion of the island by the growth of lava deltas and a steep sided central pyroclastic cone^22^. A small tsunami ~2 m high was recorded on Rakata (the southernmost and largest island of the Krakatau archipelago) during a subaerial eruption in 1981, and inferred to originate from a small flank landslide, highlighting the potential instability of the southwest flank of the volcano^18^. Apart from this event, no other tsunamis from the volcano have been reported. The most recent period of volcanic activity of AK started in June 2018 and continued into December, producing Strombolian explosions, lava flows, and ash plumes reaching altitudes of up to 5 km^23^. On Dec. 22, 2018, a major lateral collapse occurred on AK’s southwest flank, which discharged volcaniclastic material into the sea and triggered a destructive tsunami^24,25^. Based on seismic records^26^, eyewitness reports e.g.^24^, and the agreement of modelled waves with tsunami arrival times at tide gauges (Ina-COAP, 2019; see below; Fig. 1, Table 1), the collapse is estimated to have taken place at 20:55′–57′ (UTC + 7). Within 30 minutes, the tsunami flooded the coasts of west Java and southeast Sumatra, causing up to 13 m runups. 13,000 people were injured, 33,000 displaced, and thousands of buildings destroyed^1,27,28^. The tsunami struck near high tide (about 1.5 m over Mean Sea Level (MSL)) thereby increasing its impact.

**Table 1:** Lat-Lon/depth and measured^45^/simulated (0.27 km^3^ volume; granular slide) arrival times at Tide Gauges 1–4 (TG; Figs 1a, 5a–d; see Supplementary #3), and location/depth at numerical Wave Gauges 5–9 (WG; Fig. 1b).

Tide/wave Gauges (Fig. 1a,b)	Lon E. (Deg.)	Lat N. (Deg.)	Measured Arrival (s)	Simulated Arrival (s)	Depth (m)
1. TG: Serang, Jambu	105^*o*^ 50′ 27.6″	−6^*o*^ 11′ 21.5″	1920	1870	12.1
2. TG: Ciwandan	105^*o*^ 57′ 10.8″	−6^*o*^ 01′ 02.5″	2570	2500	3.7
3. TG: Kota-Angung	104^*o*^ 37′ 08.5″	−5^*o*^ 30′ 01.2″	2340	2240	3.7
4. TG: Panjang	105^*o*^ 19′ 06.1″	−5^*o*^ 28′ 08.7″	3420	3390	3.9
5. WG	105.4066^*o*^	−6.1234^*o*^	N/A	28	256.9
6. WG	105.3733^*o*^	−6.1524^*o*^	N/A	130	67.4
7. WG	105.4246^*o*^	−6.0691^*o*^	N/A	70	49.8
8. WG	105.4954^*o*^	−6.1279^*o*^	N/A	205	63.3
9. WG	105.3571^*o*^	−6.1361^*o*^	N/A	145	95.5

In simulations, AK collapse is assumed to take place at 20:57′ local time (UTC + 7). Listed depth is that in model grid G1 assuming a +1.5 m tide elevation. Difference in arrival time at tide gauges are 30–100 s, compared to the 1 min data interval. Note, as shown in Fig. 5a–d, the simulated arrival time (first crest) is closely identical for both rheologies (viscous or granular collapse); hence a single number is listed in the table. N/A: Not Applicable.

In this paper, we use high-resolution satellite imagery and aerial photography to develop a model of the AK lateral collapse scenario as a basis for numerical tsunami simulation. Tsunami simulation results are tested against time series of sea surface elevation recorded at tide-gauge, field observations of tsunami flow depth and inundation along the coasts of Java and Sumatra, and eyewitness accounts. Here, for the first time since 1883, we have an opportunity to test state-of-the-art tsunami modelling methodologies against direct observations constraining both volcanic-tsunami source parameters and observations of the generated waves. With remarkable prescience, a tsunami from a hypothetical AK collapse was modelled by^12^; the collapse in their model was to the SW, with a volume of 0.28 km^3^, and they predicted tsunami wave heights and arrival times along surrounding coastlines. Our work provides an opportunity to test the validity of this hypothetical scenario from an actual event. The results presented here constrain the style of the AK lateral collapse, providing an analogy for future events at other volcanic islands, and test current landslide-tsunami models, thus forming an important contribution towards assessing tsunami hazard from similar future events and developing improved volcanic-tsunami mitigation strategies.

## Results

### The 22^nd^ December AK collapse volume and geometry

**Figure 2:**
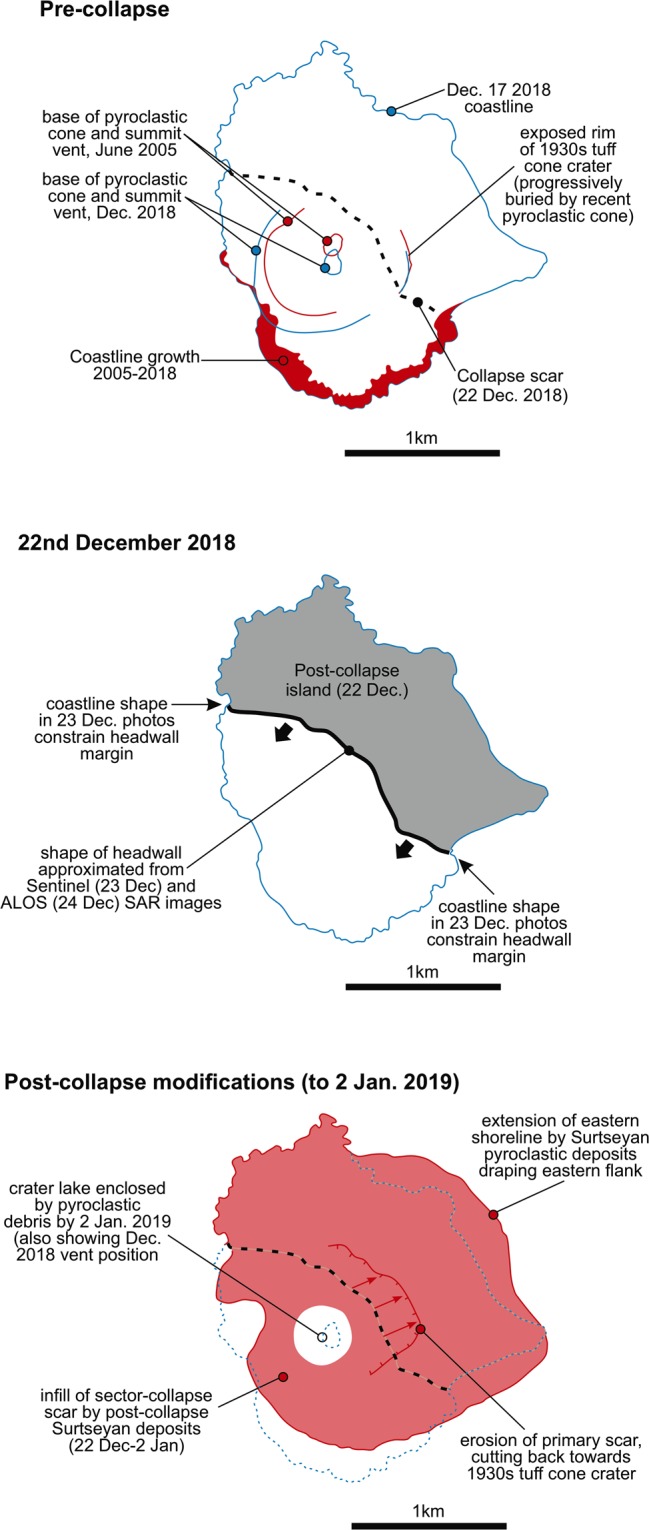
Morphological changes at Anak Krakatau spanning the December 22^nd^ 2018 lateral collapse. Features are interpreted from various satellite images^29–32^.

**Figure 3:**
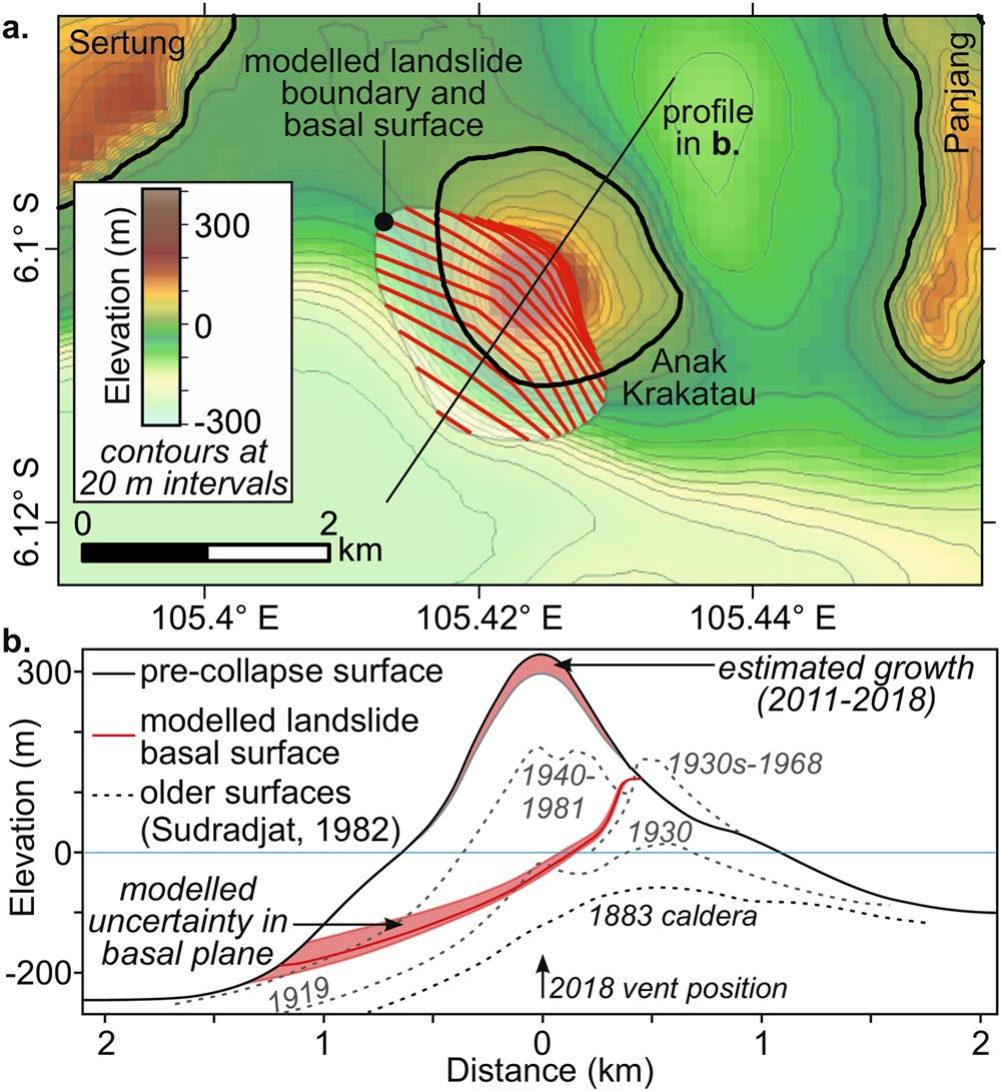
Geometry of modelled AK collapse. (**a**) Pre-collapse bathymetry and topography (colour scale and contours) of parts of Sertung and Panjang, and AK with superimposed geometry of collapsed volume (perimeter and contour levels at 20 m intervals); (**b**) Transect in SW direction (marked by black line in **a**) showing AK’s historical growth, the failure surface of the modelled most likely 0.27 km^3^ volume scenario (consistent with Fig. 2), with associated uncertainty (two additional volumes are modelled for the upper and lower failure surfaces, with a 0.22 (−20%) and 0.30 km^3^ (+10%) volume, respectively). Maps and surfaces were created by the authors with ArcMap10.2, using the dataset obtained from^12^ and the interpretation in Fig. 2.

Satellite photographs and radar images provide the key evidence from which we interpret the AK 2018 collapse geometry and volume. Oblique aerial photographs from December 23^rd^ are particularly important (Supplementary #1)^29,30^, because they unambiguously identify breaks in the pre-collapse coastline that mark the edge of the failure scarp. The photographs show a sharp, steep-sided cut of the coastal lava deltas to the NW and SE of the pre-collapse vent site. These breaks in the coast constrain the margins of the primary collapse scar on the Sentinel-1A satellite radar images from December 23^rd^ (Western Indonesian Time) and ALOS-2 images on December 24^th^ ^31,32^, which confirm the form of the landslide headwall and provide critical observations of the post-collapse coastline. Our interpretations of these radar images show that the opening angle of the headwall scar was very wide, defining a broadly linear collapse that cut behind the vent. Based on the coastlines defined in Fig. 2, the collapse reduced the subaerial area of the island by 49%. Although the photographs and radar images provide evidence on the subaerial part of the collapse, to produce such a dramatic change in the volume and shape of the island, the collapse plane evidently extended below sea level. This resulted in the submergence of the vent site, as can be seen from the intense Surtseyan explosive eruptions observed on 23^rd^ December^29,30^. In addition to the observations of the subaerial headwall shape from the satellite data, the Surtseyan explosions provide an approximate constraint on the depth of the landslide basal plane at the vent site. Historical observations suggest Surtseyan activity is characteristic of vent depths ≤50 m^33–35^. To estimate the geometry of the landslide basal plane (and hence the collapse volume), we have combined our constraints on the subaerial headwall with an assumed depth of 25 m at the vent site. We have taken a more conservative value than the 50 m depth noted above, based on the observation that debris rapidly accumulated around the vent site in the days following the collapse, reconstructing the subaerial island in this region. Finally, we have restricted the extent of the basal plane to lie within the submarine bulge of Anak Krakatau evident in bathymetry^36^ (i.e., we assume that the collapse was confined to the AK edifice, rather than cutting into older rocks of the caldera margin). These assumptions result in a concave and gently dipping form to the basal plane (Fig. 3), typical of subaerial lateral collapse scars^36,37^. Although the submarine form of the landslide scar is uncertain, a substantially smaller collapse than predicted by the above assumptions would require a sub-horizontal submerged basal plane, which is hard to reconcile with the wide collapse opening angle. We also have no evidence to infer a substantially deeper collapse scar, particularly given the rapid subaerial emergence of post-collapse debris around the vent site. We therefore consider our approximation to be appropriate given an absence of additional post-collapse bathymetric or geophysical constraints on the basal plane (noting that this plane is already likely to have been partially buried).

Because the earliest post-collapse satellite images and aerial photographs are from ~12 hours after the event, they provide no evidence on whether the collapse was a single event or occurred in stages. However, the tsunami observations, primarily the single wave train reported and absence of observed subsequent tsunamis, suggest one main phase of failure. This provides support for our numerical tsunami modelling based on a single collapse and using the headwall geometry derived from the 23^rd^ December images.

### Landslide tsunami generation and impact modelling in the near-field

Bathymetric and topographic data at an approximate 100 m resolution were obtained from the work of^12^, which was interpolated to set-up model grids (90 and 100 m resolutions; Fig. 1a,b and see details below). Comparing Fig. 1b to the pre-collapse aerial image of Fig. 1c shows that AK and the coastline of the three surrounding islands of Sertung, Panjang, and Rakata are well resolved in our grids. A simplified landslide headwall shape and basal plane geometry was defined on this grid based on the constraints outlined above. The likeliest landslide basal plane defined by these constraints is shown in Fig. 3 (solid red line), together with our estimate of the associated uncertainty (shaded red). The modelled collapse volume based on our best-estimated failure surface is calculated at 0.27 km^3^. To investigate the effects of the failure surface uncertainty on the collapse volume, we also considered and simulated collapse volumes corresponding to the upper and lower bounds of the shaded uncertainty area in Fig. 3, which yielded 10% larger (0.30 km^3^) and 20% smaller (0.22 km^3^) volumes. Our best estimated collapse volume is similar to that assumed by^12^, albeit for a different failure plane. The three failure surfaces and resulting collapse geometry and volumes were used to initialize simulations with a three-dimensional (3D) slide and hydrodynamic model of tsunami generation and propagation (“Non-Hydrostatic WAVE model” NHWAVE^38–40^; see methods), as detailed below. For each of the 3 selected collapse volumes and corresponding geometry, the 3D model was used in the near-field (Fig. 1b; grid G0, 90 m horizontal resolution with 5 boundary fitted vertical layers) to simulate the lateral collapse landslide and the corresponding tsunami generation.

As the actual collapse mechanism is unknown, for each collapse volume, simulations were performed assuming two alternative rheologies (granular material and dense viscous fluid), which were available in this model (animations of results are provided in Supplementary #2 for the 0.27 km^3^ volume scenario). Using these two rheologies allowed assessment, to some extent, of the effect on tsunami simulations of the epistemic uncertainty associated with different model physics, while the range of simulated collapse volumes allowed assessment of the effect on tsunami simulations of the aleatory uncertainty associated with selecting a failure surface and resulting collapse volume.

For each rheology, animations of model results show the 3D slide motion with or without the corresponding wave generation, which allows for easier identification of the slide in the latter case (Supplementary #2). For both rheologies, results show a rapid collapse of AK’s cone, causing a thick flow of material to the SW, which gradually fills the bottom of the caldera, together with the lateral runout of a thinner layer of slide material. With superimposed wave generation, animations show that a leading elevation wave nearly 50 m high is first generated near AK, that travels in a dominant SW direction, followed by a deeper negative elevation wave at the location formerly occupied by AK’s cone. Whereas the leading elevation wave propagates south-westward away from AK, interacting with the bathymetry and the nearby islands on which it runs up, the deep over 50 m trough near the volcano rebounds as a backwash that causes an over 40 m runup onto AK’s collapse scar. Subsequently, waves reflected from nearby islands propagate back onto AK and onto other islands. Both these reflected and initial waves cause significant runup on the most exposed parts of the three surrounding islands of Sertung to the W, Panjang to the E, and Rakata to the S (Fig. 1b,c, and Supplementary #4).

**Figure 4:**
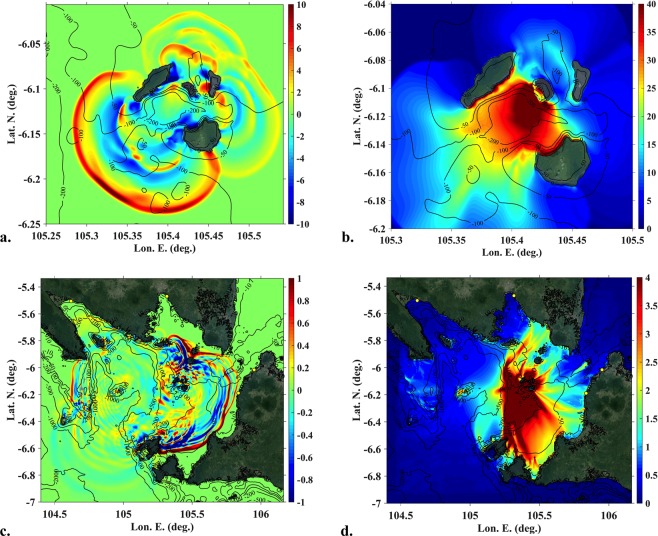
Tsunami surface elevations for AK collapse. (**a**,**b**) Instantaneous and maximum surface elevations (colour scale in meter), respectively, simulated with NHWAVE in grid G0, for the 0.27 km^3^ granular AK collapse at and up to 410 s; (**c)** Instantaneous surface elevation simulated with FUNWAVE-TVD at 1810 s in grid G1; (**d)** Envelope of NHWAVE/FUNWAVE- TVD SE up to 7,610 s. Reference level is MSL + 1.5 m (tide elevation in Marina Jambu^45^ at the time of tsunami arrival). Maps in (**a–d)** were produced by the authors based on the bathymetry/topography dataset, using MATLAB version 2017b; topography from GOOGLE EARTH georeferenced satellite images was embedded in these maps using an API key: GOOGLE EARTH, 2019 DigitalGlobe.

**Figure 5:**
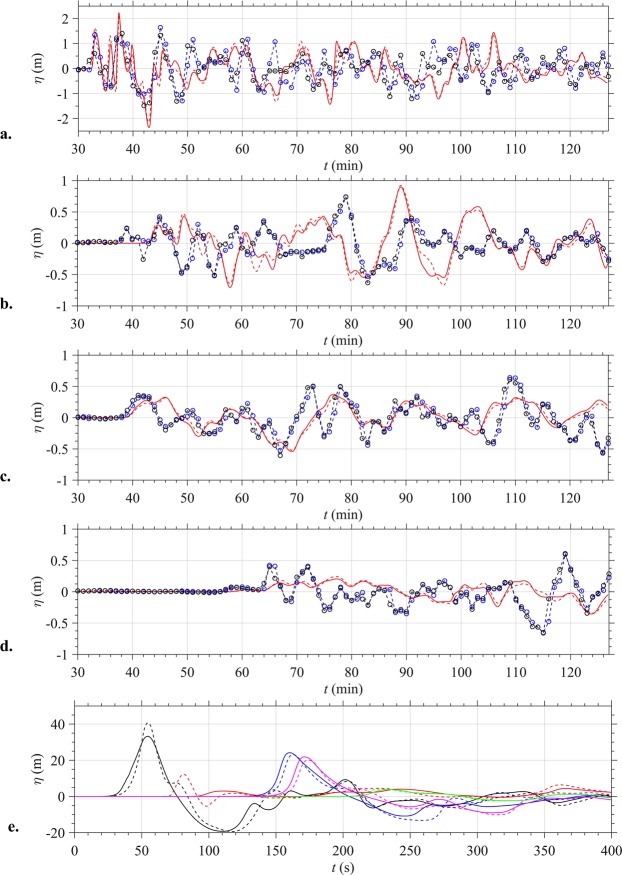
Surface elevation time series at: (**a–d)** Tide Gauges 1–4 (TG; Table 1; Fig. 1a) computed with FUNWAVE in grid G1; symbols are detided observations using 2 different sensors^45^. (**e**) Numerical Wave Gauge 5–9 (WG; Table 1; Fig. 1b) computed with NHWAVE in grid G0: (black) 5, (blue) 6, (red) 7, (green) 8, (magenta) 9. Time *t* = 0 is estimated collapse time, 20:57′ local time (UTC + 7). Computations are for (solid) viscous slide, or (dashed) granular slide.

For the 0.27 km^3^ volume likeliest scenario, Fig. 4a,b show instantaneous surface elevations computed in grid G0 at time *t* = 410 s for the granular slide, and the envelope of maximum elevation up to this time, respectively. In Fig. 4a, large leading waves (3–10 m high) are seen to propagate in all directions, but preferentially in the SW/SSE, N and ENE directions. Surface elevation time series computed for both rheologies at numerical wave gauges 5–9 (Fig. 1b; Table 1) up to 410 s (Fig. 5e) confirm these observations and show overall a good agreement with each other, particularly further away from AK (e.g., leading elevation waves in gauges 6 and 9). However, the granular slide causes significantly larger waves at gauge 7 than the viscous slide, which appears to result from the different behaviour and runout of the slide material in the northern direction (see animations in Supplementary #2). At gauge 5, close to AK in the SW direction, the leading waves are 33 and 42 m high, for the viscous and granular slides respectively. At gauges 6 and 9, further away from the volcano in the same direction, these are closer, in the 20–25 m range. At gauge 7 in the N direction, wave elevation reaches 5 and 13 m for the viscous and granular rheology, respectively, and at gauge 8 in the E direction, elevation reaches 6 and 7 m. At all numerical wave gauges, the large leading waves are followed by smaller oscillations, with a short 100–120 s period. Surface elevation time series simulated for the three volumes and the two rheologies are compared at the five numerical wave gauges in Fig. S.6d–i Supplementary #3). Overall, results are consistent with those of Fig. 5e, with a larger effect of model rheology than collapse volume (within the tested range) on wave generation in the near-field.

The surface elevation envelope of Fig. 4b shows a 25–40 m maximum runup along AK’s SW coast. On the directly exposed steep shores of the nearby islands of Rakata and Sertung runup reaches up to at least 45 m. On Panjang, which is located behind AK’s main collapse direction, due to wave refraction around the volcano (see animations in Suppl. #2), runups still reach 15–25 m. Aerial images from a drone survey on Jan. 11^th^^41^ as well as coastline changes evident in ALOS-2^42^ and Planet.com^43^ satellite images, show clear signs of large runup on the order computed with the model, on the E coast of Sertung, the W/SW coast of Panjang and NW coast of Rakata (see details in Suppl. #4). On Panjang, tree trunks are still *in-situ*, but there is very severe vegetation damage across the entire island from post-collapse, phreatomagmatic ash deposition, which obscures evidence of tsunami-related damage in the north.

### Tsunami propagation and coastal impact modelling in the far-field

The 2D model FUNWAVE-TVD (Total Variation Diminishing version of the fully nonlinear Boussinesq wave model FUNWAVE–TVD^44^; see methods) is used (Fig. 1a; grid G1, 100 m resolution) to simulate tsunami propagation from the near-field to the surrounding coasts in the far-field; the model is initialized using NHWAVE results such as shown in Fig. 4a. As for the near-filed simulations, the three different collapse volumes were simulated for both rheologies. Figure 4c shows instantaneous surface elevations computed at *t* = 1810 s for the 0.27 km^3^ granular collapse, when the leading tsunami waves are about to reach the Tide Gauge 1 at Marina Jambu^45^, on the W coast of Java (Fig. 1a; Table 1). At this time, the pattern of tsunami waves in the Sunda Straits is already quite complex, with waves propagating in many directions from their interaction with the bathymetry, the coastline, and other islands. This pattern would become even more complex as time increases, due to further multiple wave reflections within the Straits. Figure 4d shows the envelope of maximum surface elevations computed up to *t* = 7,610 s. As a result of the initial directionality of the generated wave train and bathymetric focusing, in particular by the steep linear scarp that divides the shallow eastern half of Sunda Straits from the much deeper Semangka trough to the west, wave elevations are greater in a number of narrow, preferential, directions, particularly in the S, SE, E and NE directions; the model predictions of tsunami coastal impacts are also larger at coastal sites in those directions.

For the 0.27 km^3^ volume likeliest scenario (granular case), Fig. 5a–d compare surface elevation time series computed for the viscous and granular slides to field observations made at Tide Gauges 1–4^45^ (Fig. 1a; Table 1). The surface elevation data recorded at 1 min intervals by two functioning sensors was detided using a Butterworth filter (Supplementary #3). Overall, there is a good agreement between the observed and simulated time series, particularly earlier in the time series. As summarized in Table 1, arrival times at each gauge are predicted to within 30–100 s of observations. Considering the 1 min interval of tide gauge data, this is quite a small discrepancy. Although the phase lag between simulations and observations increases with time, the trough to crest elevation of the largest waves is well captured in simulations. Additionally, each tide gauge is located within a harbor/marina or close to or behind protective coastal structures (Supplementary #3), whose complex geometry is not represented in the 100 m resolution grid G1. These surrounding structures would have induced reflections and seiching that significantly affected the recorded signal. Finally, as reported by eyewitnesses, simulations predict that multiple large waves of short period (initially 5–8 minute period) impacted the coast, with the second or later waves being the largest.

Time series of surface elevation were also computed at the four Tide Gauges for the two additional collapse volumes and each rheology. Only small differences could be observed in these far-field results between the six scenarios (Fig. S.6a–d in Supplementary #3), indicating that our predictions of the tsunami coastal impact are not very sensitive to finer details of the collapse scenario assumed for Anak Krakatau (i.e., both small changes in volume and differences in rheology). Hence, given bathymetric/topographic data and model grids, far-field tsunami impact results are quite robust. Finally, we also note that the arrival time of the first elevation wave at each tide gauge is closely identical for each scenario (Fig. 5a–d, and Fig. S.6a–d in Supplementary #3), indicating that the tsunami travel time to shore is dominated by the wave celerity, which is primarily dependent on water depth and, for these fairly short waves, to some extent, also on wave frequency. For the very large initial waves generated near the volcano, amplitude dispersion effects caused by wave nonlinearity will also affect tsunami wave celerity and thus travel time. However, results at the near-field numerical wave gauges show (Fig. 5e, and S.6e–i in Supplementary #3) that wave time series are quite similar in amplitude (and frequency) and hence effects of wave nonlinearity on tsunami celerity will also be similar. As a result of these observations, a single arrival time at the Tide Gauges was reported in Table 1 for the six simulated scenarios.

**Figure 6:**
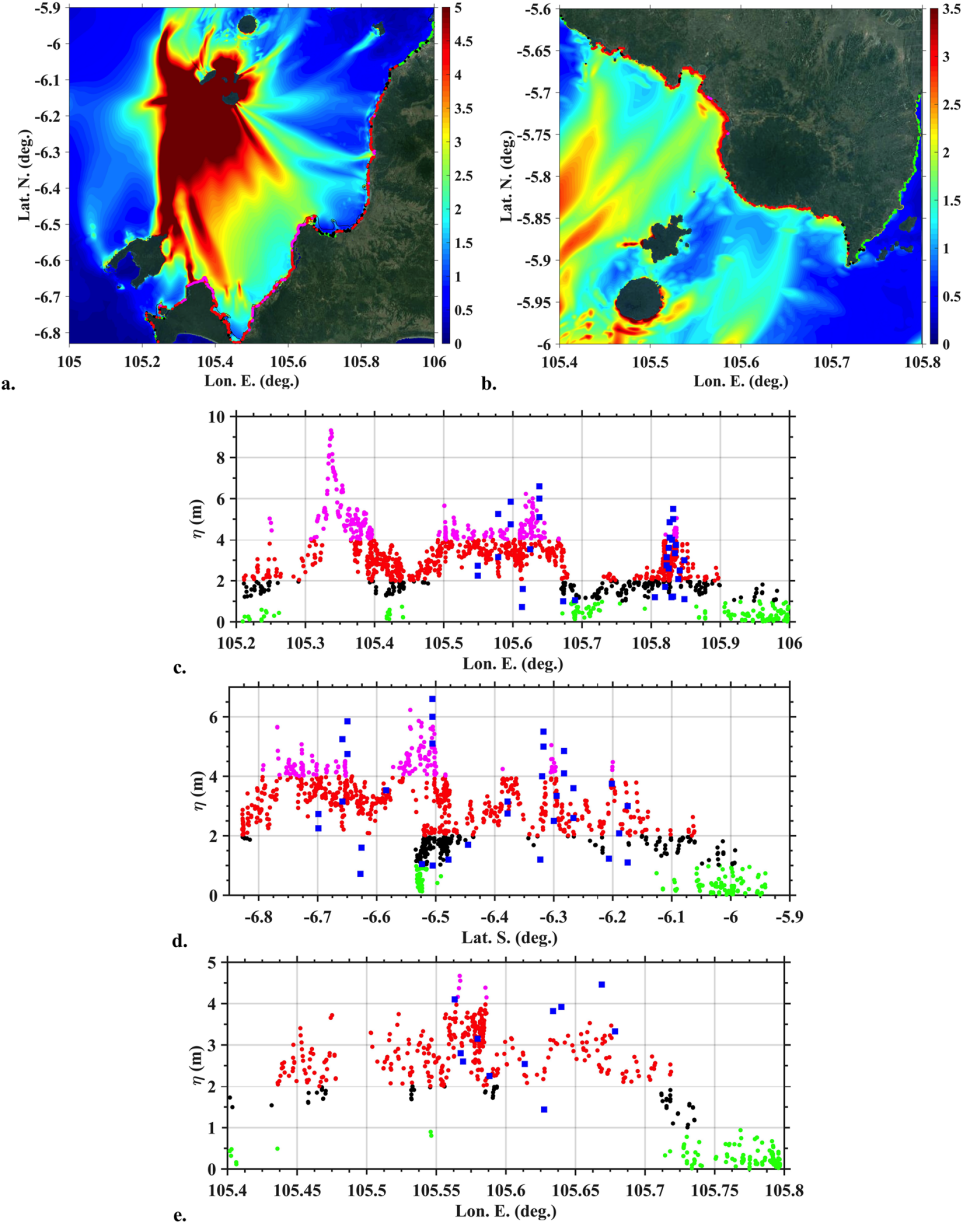
Maximum surface elevations and flow depth at the shore (along 0 m contour), simulated for the 0.27 km^3^ scenario (granular slide) based on results of Fig. 4d, in two highly impacted areas (**a**,**b**). For clarity of their drawing in (**a**,**b**), four arbitrary height classes were selected for bullets in (**c–e**). (**a**,**b**) Coastal data point locations (coloured bullets) for flow depths plotted in (**c**,**d**,**e**), respectively, shown over the maximum surface elevation (colour scale in meter). (**c**,**d)** Flow depth calculated for area (**a**) (for clarity same results were plotted as a function of Lon. or Lat.); and (**e)** area (**b**). Level datum is MSL + 1.5 m. Blue square symbols indicate data measured in field surveys^27^. Maps in (**a**,**b)** were produced by the authors using MATLAB version 2017b ; topography from GOOGLE EARTH georeferenced satellite images was embedded in these maps using an API key: GOOGLE EARTH, 2019 DigitalGlobe.

Figure 6a,b show zoom-ins of the envelope of maximum surface elevation of Figs 4d, 6c–e show the corresponding flow depths at the shore, obtained by interpolating the envelope along the 0 m contour level, for two highly impacted sections of coast in West Java and Sumatra north of Anak Krakatau. The latter results are compared to data from an initial post-event survey^27^ (note, the coordinates of surveyed data points were inferred from survey data maps using GOOGLE EARTH). Simulated and measured tsunami flow depths are found in good agreement, particularly in view of the fairly coarse model grid G1 used here. A good agreement is found in particular for the larger flow depth values measured at particular coastal sites, that result from the initial tsunami directionality and subsequent strong bathymetric focusing discussed above (note, at the time our work was performed, field data was lacking from the south-western coast of Java (Panaitan and Ujong Kulon) where the largest flow depths of 9.5 m are simulated in Fig. 6c). Additional closer zoom-ins of maximum runups are consistent with field observations and eyewitness reports (see Fig. S.3 in Supplementary #2).

## Discussion

The potential tsunami hazard from the collapse of AK was recognised before the 2018 lateral collapse^12,18^. Several factors may have predisposed the volcano for collapse of its southwest flank. First, its position on the northeast margin of the 1883 caldera has resulted in steeper submarine slopes to the southwest compared to the northeast^20,36^. Second, there has been a gradual shift in vent position during its evolution towards the southwest and the caldera interior^20^. Third, significant lava flow deltas have extended the base of AK to the west, which overlie potentially weak and altered volcaniclastic material produced by the Surtseyan activity of 1927–1960 and later hyaloclastites formed by ocean-entering lava flows as the lava deltas expanded. Finally, the very rapid growth of the volcano during the last 90 years had built a steep-sided summit cone consisting dominantly of unstable volcaniclastic material^46^.

Despite uncertainties in the volume and rheology of the collapsed material, the effects of which were estimated in our simulations based on six scenarios, the good correspondence between the predicted and observed wave characteristics in the far-field at the coast, flow depths, and runups, confirms the relevance of the proposed geometric model for the collapse. In particular, the modelling is consistent with a tsunami source of 0.22–0.30 km^3^, as inferred from our interpretation of post-collapse imagery (Figs 2, 3). Combined with the observation of a single phase of tsunami inundation, our results support an interpretation of an *en masse* failure of the SW flank of Anak Krakatau, rather than a more piecemeal process, and suggest that this was necessary to generate the observed tsunami. Our results also suggest that significant portions of the submarine flank of AK were involved in the landslide. As identified previously by^12^ the mechanism of failure (for example, whether a landslide occurs in a multi-stage retrogressive process) is important in determining the characteristics of the generated tsunami, particularly in the near-field. In this instance, our results confirm that failure had to be sufficiently rapid to represent a single tsunami source, which is consistent with the observed tsunami wave train. In addition, based on our simulations, it is not necessary to invoke any other possible tsunami-generating mechanism, such as a lateral blast or submarine explosion, to explain the magnitude and intensity of the tsunami.

It remains unclear what role eruptive activity had in triggering the lateral collapse. The collapse occurred several months into an eruption phase that was relatively intense but comparable in style to the activity that has characterised preceding decades, and there was no clear indication of increasing or anomalous activity in the days or weeks preceding the collapse^23,25^. Although a major change in eruptive behaviour, in the form of intense phreatomagmatic explosions, occurred after the collapse, current observations cannot identify whether this change was a response to the collapse, or whether an incipient change in behaviour occurred in the run-up to the collapse and may thus have acted as a trigger for failure. Given that there are examples of volcanic lateral collapses which were not associated with magmatic eruptions^47^, it is plausible that the collapse was simply associated with a critical instability arising from the long-term rapid growth and local topographic and geological characteristics of AK.

Numerical simulations of six collapse scenarios, including both slide and tsunami generation, and propagation to shore, were performed that covered the range of uncertainty in estimated collapse volume, for two different slide rheologies (granular material and dense viscous fluid). Overall, results showed that waves generated in the near-field were quite similar for each scenario, particularly in the dominant direction of propagation (SW/SSE) (Figs 5e, S.6e–i). This similarity was even more pronounced for far-field results, as time series of surface elevations simulated at the four tide gauges were very close for each scenario (Figs 5a–d, S.6a–d). This “loss of memory” of the details of the tsunami source, as a tsunami propagates away from it is expected^48^. Nearshore, this effect is even more pronounced as a result of the strong bathymetric control on the refraction and shoaling of tsunami waves^48^. Given the relatively minor effect of both the collapse volume and slide rheology on the far-field tsunami impact (a feature already pointed out in the hypothetical scenarios simulated by^12^), simulation results were mostly shown here in detail and compared to field data for the likeliest collapse scenario (0.27 km^3^), assuming a granular rheology. This also implies that the limited choice of two model rheologies was adequate to estimate the far-field tsunami impact. A good agreement with field data was observed for the arrival time and elevation of the first few waves at tide gauges, as well as for the flow depth at the shore, which confirmed the relevance of the estimated collapse scenario(s) and the accuracy of numerical simulations with the NHWAVE/FUNWAVE-TVD model suite. The latter implies that a similar modelling approach could be applied to estimate tsunami hazard from future hypothetical volcanic collapses at other locations. Although these models have been well validated and benchmarked against laboratory data and some field case studies, to date, they had not been applied to a well-documented large scale volcanic collapse such as AK’s.

One of the most significant developments following the AK eruption is the rapid post-collapse regrowth of the volcanic edifice by Surtseyan activity, which in the space of a few weeks significantly modified the morphology of the collapse scar. The rapidity of AK’s rebuilding following the collapse signals the potential for future collapse. Seawater gained access to the vent in the immediate post-collapse period, generating distinctive Surtseyan ash plumes and in a sense resetting the evolution of the volcano back to the type of activity occurring in the 1930–1960 period. The rebuilding has already led to an enclosed crater lake, and it is likely that further activity will rapidly establish a new cone, with a transition back to magmatic eruptions and lava effusion. The southwest submarine flank is again being supplied with unconsolidated volcaniclastic material and if lava flows extend the coast in this direction, they will overlie these weaker lithologies, as was the situation prior to the December 2018 failure. AK’s rapid regrowth emphasizes the need to monitor its activity by the acquisition of satellite data, as well as marine surveys to better determine submarine bathymetry. In both volume and volcano-tectonic setting, the AK event is broadly similar to lateral collapse-generated tsunamis at other arc volcanoes including Oshima-Oshima 1741, Unzen 1792, Ritter Island 1888, and Harimkotan 1930^5,7,49–52^. These historical events suggest that tsunami-generating volcanic landslides >0.1 km^3^ have a global recurrence interval of less than 100 years. Although there are likely to be differences in collapse precursors, dominant lithologies and the style of landslide emplacement between all events e.g.^16,51^, the AK lateral collapse is a modern equivalent of these historical events. As such, it is significant both in improving our understanding of the mechanisms of lateral collapse and as a benchmark against which current landslide-tsunami models can be tested and developed. The AK lateral collapse highlights the major hazard from this volcanic process, both in the impact of the initial landslide and, in island or coastal settings, from the associated tsunami generation.

If the regrowth of AK continues, there is a possibility of future collapses to the southwest and potential tsunami generation. However, the precursors to lateral collapses remain poorly understood, limiting the prospects for forecasting their timing. Given the short tsunami travel time from the AK volcanic source to the adjacent coastlines, mitigating against tsunami impacts after generation based on standard buoy-based measurements is a major challenge. Unlike earthquake-triggered tsunamis, which can be more easily detected, and hazard warnings issued to adjacent coastal communities, volcanic lateral collapses will continue to be a surprise, with the potential for causing significant loss of life and property destruction. One promising avenue for early detection of tsunamis from such non-seismic sources is the use of shore-based high frequency radars, combined with tsunami detection algorithms based on simulations of potential tsunami scenarios cf.^53^, and literature reviews therein. Following the premonitory study of^12^, the present research confirms that state-of-the-art numerical models can accurately simulate the potential hazard from volcanic induced tsunamis. Hence, realistic lateral collapse scenarios could be simulated for future volcanic events, at AK or elsewhere, and serve as a basis for developing tsunami hazard assessment maps and new types of tsunami detection and warning systems.

## Methods

### Numerical models and grids

We use a suite of hydrodynamic models for 3D landslide tsunami generation (NHWAVE)^38–40^ and 2D propagation (FUNWAVE-TVD)^44^. These models have been extensively validated through benchmarking against laboratory and field data, as well as in the context of operational tsunami hazard assessment, for hypothetical^38,39,53–56^ or actual^57–60^ landslide tsunamis and other events. Much of this past work was performed in the US in parallel with or under the auspice of the National Tsunami Hazard Mitigation (NTHMP) program, including studies of potential volcanic flank collapses in the Canary Islands^11,48^ (see also^9^ for initial work on this event).

NHWAVE simulates 3D wave generation by slides, including frequency dispersion effects (vertical acceleration/non- hydrostatic effects), using only a small number of vertical, boundary fitted, *σ* -layers. Slides are modelled as an additional (depth-averaged) layer of subaerial and/or underwater material whose motion is fully coupled to that of the surrounding water. This layer can be of arbitrary geometry and also simulate non-hydrostatic effects^61^, with the slide material being either represented as a dense Newtonian fluid^39^ or a saturated granular medium with intergranular and basal stresses governed by Coulomb friction^40,55^. Both of these *rheologies* were used in the present work. FUNWAVE-TVD is a fully nonlinear and dispersive 2D Boussinesq-type model used (and initialized) here to propagate the initial near-field tsunami generated with NHWAVE to the far-field. FUNWAVE-TVD has been applied and validated for some actual tsunami case studies^57–60^.

The 100 m resolution bathymetric and topographic data was interpolated to set-up model grids, to which +1.5 m was added, representing the tide elevation at Marina Jambu (tide gauge 1; Fig. 1a) at the time of tsunami arrival: (i) a Cartesian grid G1 used in FUNWAVE-TVD, with a 100 m horizontal resolution (Fig. 1a; 1950 by 1840 cells), and (ii) a 3D grid G0, with a 90 m horizontal resolution and 5 *σ* -layers, over a smaller footprint nested within G1 (Fig. 1b; 360 by 300 cells). Based on AK’s collapse geometry described in Fig. 3 the likeliest collapse volume, and its lower and upper bounds (within the estimated uncertainty) are computed at 0.27, 0.22 and 0.30 km^3^, respectively in NHWAVE grid G0 (Supplementary #2 provides 3D images of the pre- and post-collapse discretized topography and bathymetry in grid G0).

### Tsunami simulations

Following initial sensitivity analyses performed with another model (TSUNAMI-SQUARE^62^), six collapse cases were simulated with NHWAVE in grid G0 (Fig. 1), for 0.22, 0.27, and 0.30 km^3^ volume of slide material (Fig. 3) represented: (i) either as a Newtonian fluid of density *ρ*_*c*_ = 1, 900 kg/m^3^ and kinematic viscosity *φ*_*c*_ = 0.5 m^2^/s; or (ii) a granular medium with *ρ*_*c*_ = 1, 900 kg/m^3^ for the solid part, an internal friction angle *φ*_*ic*_ = 10°, a basal friction angle *φ*_*bc*_ = 2° (similar to that used by^15^, and a 40% porosity; with this data, assuming a water density *ρ*_*w*_ = 1, 025 kg/m^3^, the average density of the granular medium is *ρ*_*ac*_ = 1, 550 kg/m^3^). NHWAVE was run up to 410 s, the time when the generated tsunami approaches the boundary of grid G0 (Fig. 4a). Then surface elevation and horizontal velocity (calculated at the required depth *z* = −0.531 *h*) were interpolated to initialize FUNWAVE-TVD simulations in grid G1^55^, which were run for another 7,200 s (Fig. 4c,d). Time series of surface elevations shown in Fig. 5 (and Fig. S.6) are computed at 5 numerical wave gauges in NHWAVE and 4 tide gauges in FUNWAVE-TVD (Table 1).

## Supplementary information

Grilli_etal_AK_supplementary_revised_updated_rerevised1

Krakatau_slide3D_visc

Krakatau_wave_slide3D_visc

Krakatau_slide3D_gran

Krakatau_wave_slide3D_gran

## Data Availability

The FUNWAVE-TVD and NHWAVE models used here (developed by some of the authors) are open source and available on github.com. All data is available from references cited in the text, except for unprocessed raw input-output model results, which have been archived and the authors can make available upon request.
