# Supplementary material for: Modelling of the tsunami from the December 22, 2018 lateral collapse of Anak Krakatau volcano in the Sunda Straits, Indonesia
Source: Sci Rep. 2019 Aug 16;9:11946. doi: 10.1038/s41598-019-48327-6 (PMC6697749; doi:10.1038/s41598-019-48327-6)
Supplement: Supplementary file 1 — Grilli_etal_AK_supplementary_revised_updated_rerevised1 [file 41598_2019_48327_MOESM1_ESM.pdf]

Supplementary Information for

Modelling of the tsunami from the December 22, 2018 lateral collapse of Anak Krakatau volcano in the Sunda Straits, Indonesia

Stephan T. Grilli[1,2,*], David R. Tappin[3,4,+], Steven Carey[2,+], Sebastian F.L. Watt[6,+], Steve N. Ward[5,+], Annette R. Grilli[1,+], Samantha L. Engwell[3,+], Cheng Zhang[7,+], James T. Kirby[7,+], Lauren Schambach[1,+], and Muslim Muin[8,+]

[1]Department of Ocean Engineering, Univ. of Rhode Island (URI), Narragansett, RI, USA
[2]Graduate School of Oceanography, University of Rhode Island URI), Narragansett, RI, USA
[3]British Geological Survey (BGS), Nottingham, UK
[4]University College London (UCL), London, UK
[5]University of California Santa Cruz (UCSC), Santa Cruz, CA, USA
[6]School of Geography, Earth and Environmental Sciences, University of Birmingham (UB), Birmingham, UK
[7]Center for Applied Coastal Research, University of Delaware (UD), Newark, DE, USA
[8]Bandung Institute of Technology, Bandung, Indonesia
[*]grilli@uri.edu

<u>Supplementary File #1</u> – Extended constraints on the Anak Krakatau 22nd December 2018 lateral collapse geometry

The principal sources of information on the shape and volume of the Anak Krakatau lateral collapse are satellite optical images from before the collapse; satellite radar images collected in the days immediately following the event; and oblique aerial photographs, particularly those taken on 23rd December (the day after the collapse). Our interpreted dimensions of the failure mass are based on these, alongside other volcanological interpretations of the growth history and eruptive activity of Anak Krakatau.

S1.1. The pre-collapse edifice

The lateral dimensions of the pre-collapse Anak Krakatau edifice are well constrained from satellite images and aerial photographs taken over the past twenty years, which document the growth of Anak Krakatau (Fig. 2). Over this period, growth involved ~100 metres of extension of the coastline to the south and south-east, by lava delta progradation, and an increase in diameter of the central pyroclastic cone, evident from the change in surface morphology that highlights the break in slope at the base of the cone. The pre-collapse height of Anak Krakatau is less well determined. Digital elevation models (at 1-arc second resolution) suggest a summit height of ~250 m in 2011 (ASTER; 17/10/2011)[1], and ~260 m in 2014 (SRTM; 23/9/2014)[1]. However, in both of these the summit shape is smoothed into a domed profile, and doesn't resolve the cratered form evident in satellite images, suggesting that the summit height may be underestimated. In addition, satellite images clearly show lateral growth by eruptive activity since 2014, likely

to have been accompanied by an increase in summit elevation. The map in *Abdurachman et al.* (2018) suggests a summit height of 310 m. A height in excess of 300 m is also suggested by media reports following the collapse (e.g., 338 m referred to in[2]), although the basis of these measurements is unclear. Photographs from the summer of 2018[3] show that the active vent was south-west of the cratered summit of the 2014 cone, and had formed a rounded pyroclastic cone on this side that projected well above the crater rim of the older cone. Based on this, we have estimated that the summit of Anak Krakatau had a maximum elevation of 330 m prior to its collapse, but note that this is not precisely constrained, and may have been closer to 300 m.

For the tsunami modelling, we used a 100 m resolution grid based on *Giachetti et al.* (2012), but increased the elevation of the Anak Krakatau pyroclastic cone to 330 m (by multiplying elevations by a factor of 1.1), as indicated in Fig. 3. The relatively low resolution of the subaerial island in this grid gives a smoothed coastline and edifice shape relative to the ASTER/SRTM digital elevation models, but there is little difference in the volume of the edifice or of the subaerial landslide mass. Given other uncertainties in the landslide basal plane and total volume (discussed below), we suggest that the simplification in the shape of the subaerial volcano is not likely to be a significant source of error in the tsunami source model, particularly compared to uncertainty in the pre-collapse geometry of the submarine slopes of the volcano.

S1.2. The subaerial landslide headwall

Determining the shape of the subaerial lateral-collapse headwall is key to obtaining an accurate estimate of the primary landslide volume, and particularly how much of the 330-m high pyroclastic cone was involved in the collapse. In addition to the coastline shape observed in Sentinel-1A and ALOS-2 SAR images from the 23rd and 24th December (Fig. 2, S.1.), aerial photographs taken on 23rd December provide important constraints and the northern and southern margins of the collapse scar. Images posted on several websites of Surtseyan explosions on 23rd December also show the remains of the former coastline[4,5,6,7]. This indented coastline was the result of multiple overlapping lava-delta lobes, and the margins of the scar are easily located relative to this complex coastal shape. The shape of the headwall interpreted from these images is shown in Fig. 2 and is the basis of our estimated landslide geometry. The shape has been simplified in the smoothed 100-m grid used for our simulations, while maintaining the same overall dimensions.

The Sentinel-1A image from 23rd December shows a relatively linear, wide-angled collapse scar, but it is ambiguous whether this entirely encompasses the pre-collapse pyroclastic cone of Anak Krakatau. The bright areas in the central part of the collapse scar suggest that parts of the NE flank of the cone may remain in situ, while the southernmost part of the 1930s tuff cone crater also appears to be present. Thus, although the scar clearly cut beyond the active vent site and removed the majority of the cone, a residual part may have remained in place. This remaining material is likely to have been highly unstable, and subsequent images suggest erosion of the collapse scar to the east and north-east, cutting back towards and possibly beyond the line of the 1930s tuff cone crater (see interpretation in Fig. 2). Note however that interpretation of this early radar image is complicated by the significant radar reflectivity of the eruption plume, which partly obscures the eastern part of the island, and also the potential for radar signals to be backscattered by pyroclastic material that may have accumulated around the vent site (the potential presence of floating

debris is suggested by aerial photographs taken on 23rd December, noted above[4,5,6,7]). Nevertheless this first radar image is the most useful one, because later satellite images also highlight the very rapid post-collapse infilling of the primary collapse scar, and growth of the island coastline to the east (Fig. 2). As a result, satellite images from after 24th December are a poor guide to the initial collapse geometry.

S.1.3. Constraints on the landslide basal surface

The observation of Surtseyan explosions the day after the lateral collapse indicates that the vent site was submarine, and that the headwall slope was thus relatively steep (cutting from ~150 m (the estimated height of the post-collapse scar) to beneath sea level, over a lateral distance of ~300 m). As discussed in the main text, the relationship between Surtseyan explosion styles and water depth is not well characterized, but historical observations of similar behaviour occurred at vent depths ≤50 m (*Moore*, 1985; *Belousov and Belousova*, 2000; *Cole et al.*, 2001). The location of the post-collapse crater lake, which formed as the island re-grew through to early January 2019, coincides with that of the pre-collapse vent: we thus infer that the same conduit remained active throughout the event. From this, we interpret that the collapse cut the active conduit at a depth of < 50 m but beneath sea level, and have adopted a nominal depth of 25 m in our estimated basal surface geometry. From this, it is clear that there must have been a submarine component of the landslide, but there is limited evidence on which to estimate the extent of this.

The shape of the submarine edifice of Anak Krakatau is evident from bathymetry in *Deplus et al.* (1995), showing that the SW flank of the cone overlaps with the margin of the 1883 caldera, forming a topographic bulge on the caldera rim. We have assumed that the initial failure of the landslide mass did not extend beyond or below the base of the Anak Krakatau edifice, and have thus selected a shape that continues from the subaerial scar, but remains within the contours of the SW submarine flank to define a smooth symmetrical form. The shape derived from this is shown in Fig. 3. If the foot of the landslide is to remain within these contours, it is clear that the steep headwall gradient must transition into a much more gentle basal surface, defining a concave basal landslide geometry that is typical of volcanic lateral collapses in general (e.g., *Glicken*, 1996; *Watt et al.*, 2014). The precise shape of this is poorly defined. A more conservative, shallower basal plane would result in a reduced landslide volume (a reduction of 20% is indicated in Fig. 3).

Although bathymetric information from Anak Krakatau is fairly limited, the growth of the island has been repeatedly surveyed since 1930, and *Sudradjat* (1982) provides a cross section that indicates this growth pattern. Surfaces from this cross section are shown in Fig. 3 and demonstrate that our estimated landslide surface falls within the Anak Krakatau edifice, rather than cutting into older caldera-wall rocks. The surfaces also suggest that the landslide headwall did not cut back as far as the northeast rim of the pre-1960 tuff cone crater, which increased in elevation until the 1960s, when the Anak Krakatau vent became subaerial and phreatomagmatic activity ceased.

S.1.4. Uncertainties and limitations

Although the information summarised above constrains the shape of the initial landslide headwall and also highlights post-collapse modifications of the subaerial island, it is important to recognise limitation in these constraints. The shape of the central part of the headwall, and particularly its gradient, cannot be precisely constrained. The Surtseyan

post-collapse activity shows that there must have been a submarine component to the landslide, but this same activity rapidly obscured and infilled parts of the scar, and may also have been accompanied by erosion of the steeper parts of the headwall. This post-collapse modification means that the high-resolution optical images taken from December 29 onwards do not help constrain the primary landslide scar geometry. To better estimate the submarine surface of the landslide is likely to require geophysical surveying.

The available images also do not allow us to determine if the collapse occurred in a single stage or in a piecemeal manner. However, both wave observations and seismic records suggest a rapid single-stage collapse, or very closely spaced stages, such as occurred during the collapse of Mount St Helens in 1980 (*Glicken*, 1996), and as inferred from the single observed tsunami wave train generated by the Ritter Island collapse, in 1888 (*Ward and Day*, 2003; *Day et al.*, 2015). Our model results also imply that only a collapse of this type would be capable of generating the observed magnitude of tsunami. Additionally, our observations can't discern if there was an explosive or eruption-associated component to tsunami generation, in addition to the mass movement. However, we emphasise that our model results currently suggest that such a process is not required as source component of the tsunami, given uncertainties in the collapse volume.

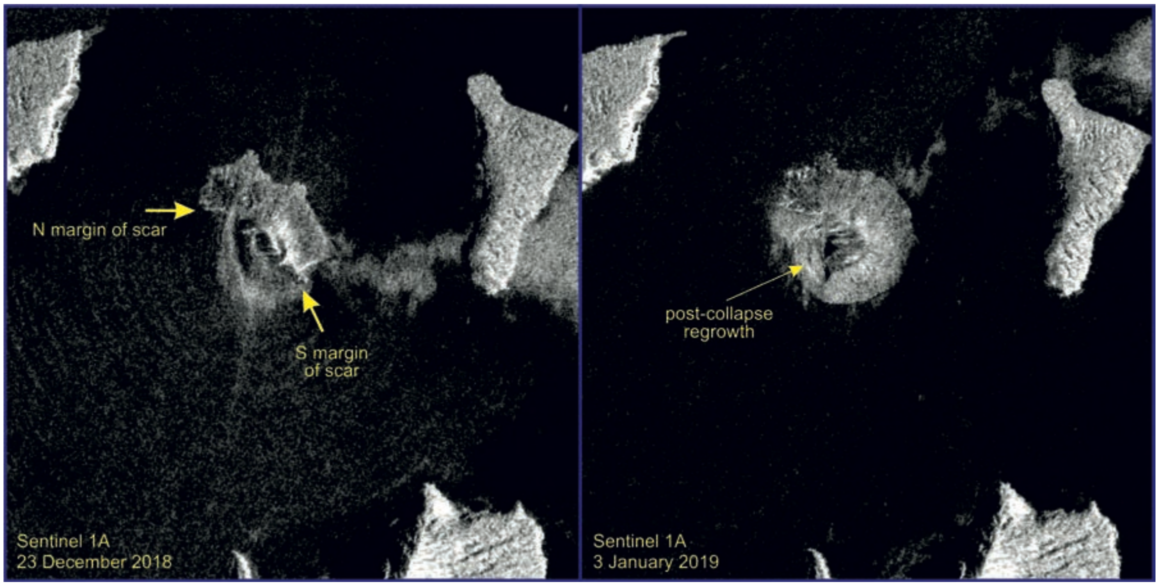

Figure S.1. Satellite images constraining the primary Anak Krakatau collapse scar. The margins of the scar have been interpreted in conjunction with aerial photographs published on several online sources following the collapse[4,5,6,7]. The left panel shows a Sentinel-1A SAR satellite image from 23 December. The dramatic changes in the coastline that occurred within ten days of the collapse are evident in the 3 January Sentinel-1A SAR image (right). Images contain modified Copernicus Sentinel data [2019] processed by Sentinel Hub[8].

References

Abdurachman, M., Widiyantoro, S., Priadi, B., and Ismael, T. 2018. Geochemistry and structure of Krakatoa volcano in the Sunda Strait, Indonesia. *Geosciences, 8*, 111.

Belousov, A. and M. Belousova 2000. Formation of partially submerged tuff ring during the 1996 sublacustrine surtseyan eruption in Karymskoye lake, Kamchatka, Russia. Terra Nostra 2000/6: International Maar Conference, pp. 42-52.

Cole, P.D., J.E. Guest, A.M. Duncan and J.-M. Pacheco 2001. Capelinhos 1957-58, Faial, Azores: deposits formed by an emergent surtseyan eruption. *Bull. Volcanol.*, *63*, 204-220.

Day, S., Llanes, P., Silver, E., Hoffmann, G., Ward, S., Driscoll, N. 2015. Submarine landslide deposits of the historical lateral collapse of Ritter Island, Papua New Guinea. *Mar. Petrol. Geol, 67*, 419-438.

Giachetti, T., Paris, R., Kelfoun, K. and Ontowirjo, B. 2012. Tsunami hazard related to flank collapse of Anak Krakatau volcano, Sunda Straits, Indonesia. In J. Terry and J. Goff (eds), *Natural Hazards in the Asia-Pacific Region: Recent Advances and Emerging Concepts*, Geological Society, London, Special Publications (vol. 361, pp. 79-90). London: The Geological Society of London.

Glicken, H. 1996. Rockslide-debris avalanche of May 18, 1980, Mount St. Helens volcano, Washington. U.S. Geological Survey, Open-file Report 96-677, 90 pp.

Ina-COAP 2018. Tide Gauge data in Indonesia. http://tides.big.go.id/las/UI.vm

Moore, J.G. 1985. Structure and eruptive mechanisms at Surtsey Volcano, Iceland. *Geol. Mag.*, *122*, 649-661.

Sudradjat, A. 1982. The morphological development of Anak Krakatau volcano, Sunda Straits. *Geology of Indonesia*, *9*, 1-11.

Ward, S.N., S. Day 2003. Ritter Island Volcano – lateral collapse and the tsunami of 1888. *Geophy. J. Intl., 154*, 891-902.

Watt, S.F.L., P.J. Talling and J.E. Hunt 2014. New insights into the emplacement dynamics of volcanic island landslides. *Oceanography*, *27*, 46-57.

Online images retrieved in January 2019 from:

[1] https://earthexplorer.usgs.gov/

[2] http://en.tempo.co/read/922253/5-facts-about-mount-anak-krakatau

[3] https://earthobservatory.sg/blog/child-krakatoa-awakes

[4] https://kumparan.com/@kumparannews/foto-udara-situasi-terkini-gunung-anak-krakatau-1545561798885566899

[5] https://www.idntimes.com/news/indonesia/sunariyah/beberapa-menit-sebelum-tsunami-banten-gunung-anak-krakatau-meletus

[6] https://foto.tempo.co/read/69191/kondisi-waspada-begini-erupsi-gunung-anak-krakatau-dari-udara

[7] http://aceh.tribunnews.com/2018/12/24/foto-foto-letusan-gunung-anak-krakatau-sehari-setelah-tsunami-menerjang-banten-dan-lampung

[8] https://apps.sentinel-hub.com/eo-browser

Supplementary File #2 – Details of NHWAVE and FUNWAVE-TVD simulation results

a.

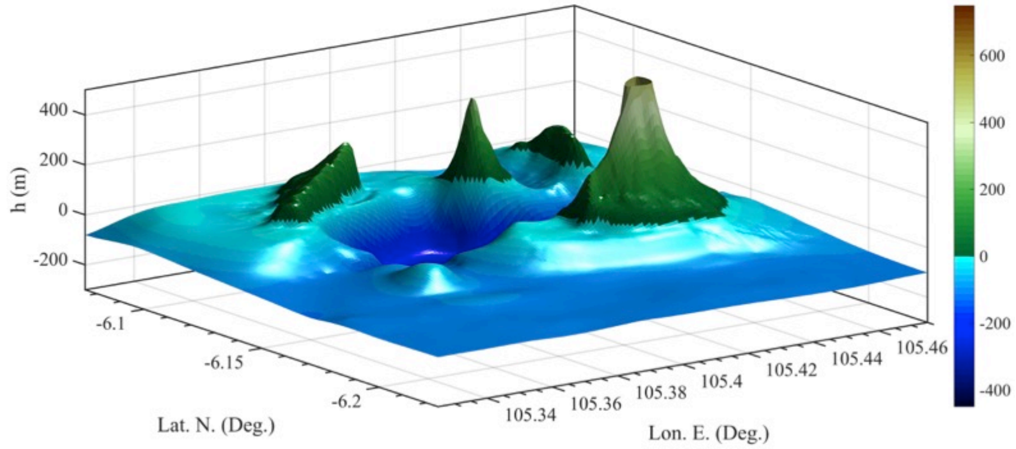

a.

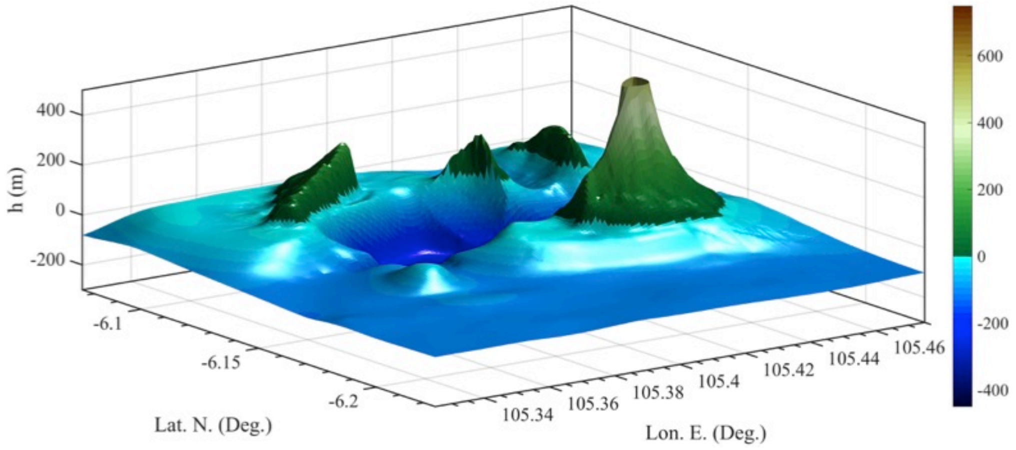

Figure S.2. (**a.**,**b.**) 3D view of AK's pre-and post-collapse (based on Fig. 3) bathymetry and topography, respectively (colour scale in meter; a +1.5 m tide elevation was added), interpolated onto NHWAVE grid G0 (Fig. 1). The islands of Sertung (W), Panjang/Kitjil (E), and Rakata/Krakatau (S) are also visible. The volume removed from AK in (**b**.) is 0.27 km^3 (likeliest scenarios with granular slide rheology; Fig. 3)

Figure S.2 shows 3D views computed based on Fig. 2 of the pre-and post-collapse bathymetry and topography interpolated over NHWAVE grid G0 (see also Suppl. #1); and Figure S.3 shows zoom-ins of the maximum envelope of surface elevations computed in Fig. 4d, based on NHWAVE and FUNWAVE-TVD results at different locations along the west coast of Java and the southeast coast of Sumatra where field surveys were performed (TDMRC, 2019). Both figures are shown for the 0.27 km^3 likeliest volume, with granular slide rheology, as results of all 6 cases simulated are qualitatively similar (and quantitatively close) results (see discussion in the main text).

Note that, besides using NHWAVE and FUNWAVE-TVD, some preliminary simulations and sensitivity analyses to the AK collapse parameters were performed using

TSUNAMI-SQUARES, a simplified but more computationally efficient model simulating submarine/subaerial slides and edifice collapse, based on momentum exchange between discrete elements (e.g., Xiao et al., 2015).

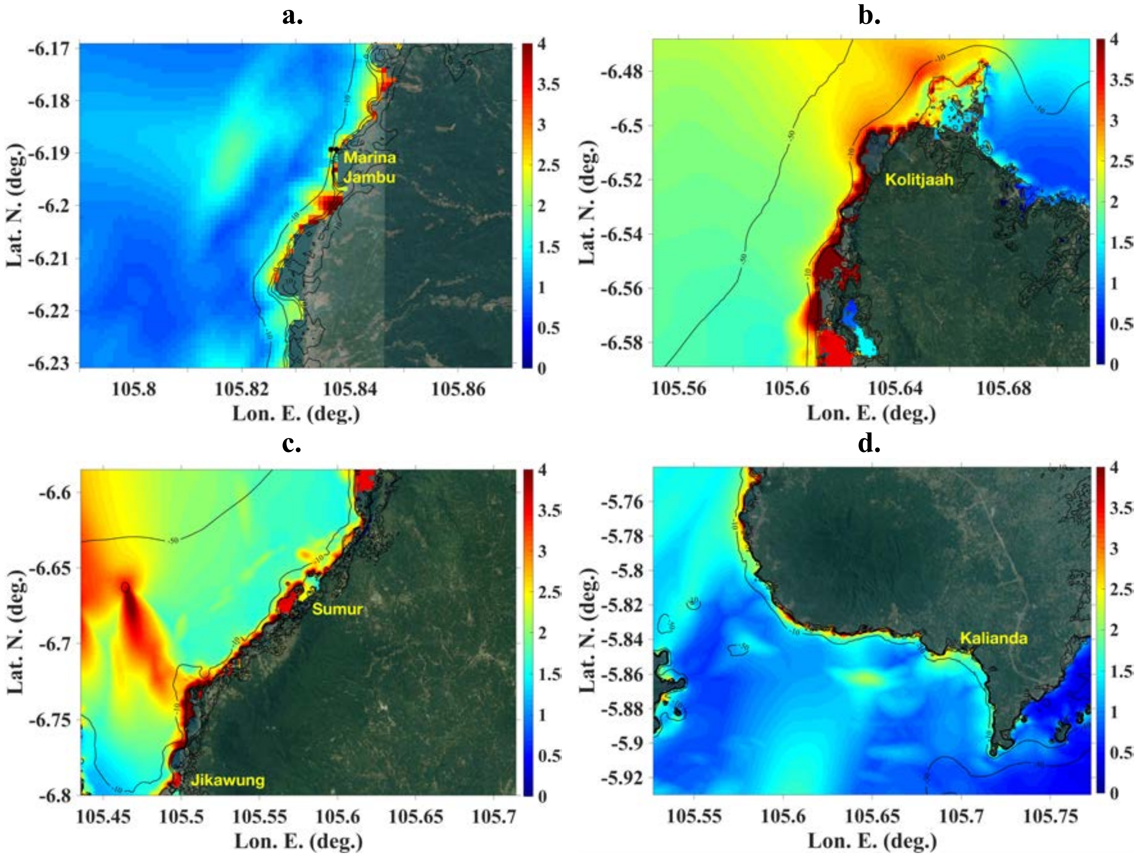

Figure S.3. Zoom-in of Fig. 4d, showing maximum elevations simulated at different locations along the west coast of Java (**a.-c.**) and southeast coast of Sumatra (**d.**) for the granular AK collapse with 0.27 km^3 volume (likeliest scenario; Fig. 3). Reference level is MSL + 1.5 m. Maps in **a.** to **d.** were produced by the authors based on the bathymetry/topography dataset, using MATLAB version 2017b; topography from GOOGLE EARTH georeferenced satellite images was embedded in these maps using an API key: GOOGLE EARTH, 2019 DigitalGlobe.

Finally, 3D animations of NHWAVE simulation results, for viscous and granular slide scenarios are shown in files:

(i) Krakatau_slide3D_gran.mp4 for the granular slide;
(ii) Krakatau_slide3D_visc.mp4 for the viscous slide;
(iii) Krakatau_wave_slide3D_gran.mp4 for the granular slide with wave generation; and
(iv) Krakatau_wave_slide3D_visc.mp4 for the viscous slide with wave generation.

These animations clearly show where the slide material runs out onto the seafloor, as well as near-field wave generation, propagation and interaction with the surrounding islands.

References

Xiao, L., Ward, S.N. and Wang, J. (2015). Tsunami Squares Approach to Landslide-Generated Waves: Application to Gongjiafang Landslide, Three Gorges Reservoir, China. Pure Appl. Geophys. 172, 3639-3654, doi:10.1007/s00024-015-1045-6.

TDMRC (Tsunami Disaster and Mitigation Research Center) Post Sunda Strait Tsunami Survey. http://tdmrc.unsyiah.ac.id/the-latest-update-from-post-sunda-strait-tsunami-survey/. Accessed Jan. 8, 2019 (2019).

Supplementary File #3 – Details of tide gauge locations, data analysis, and tide and wave gauge results sensitivity to collapse volume

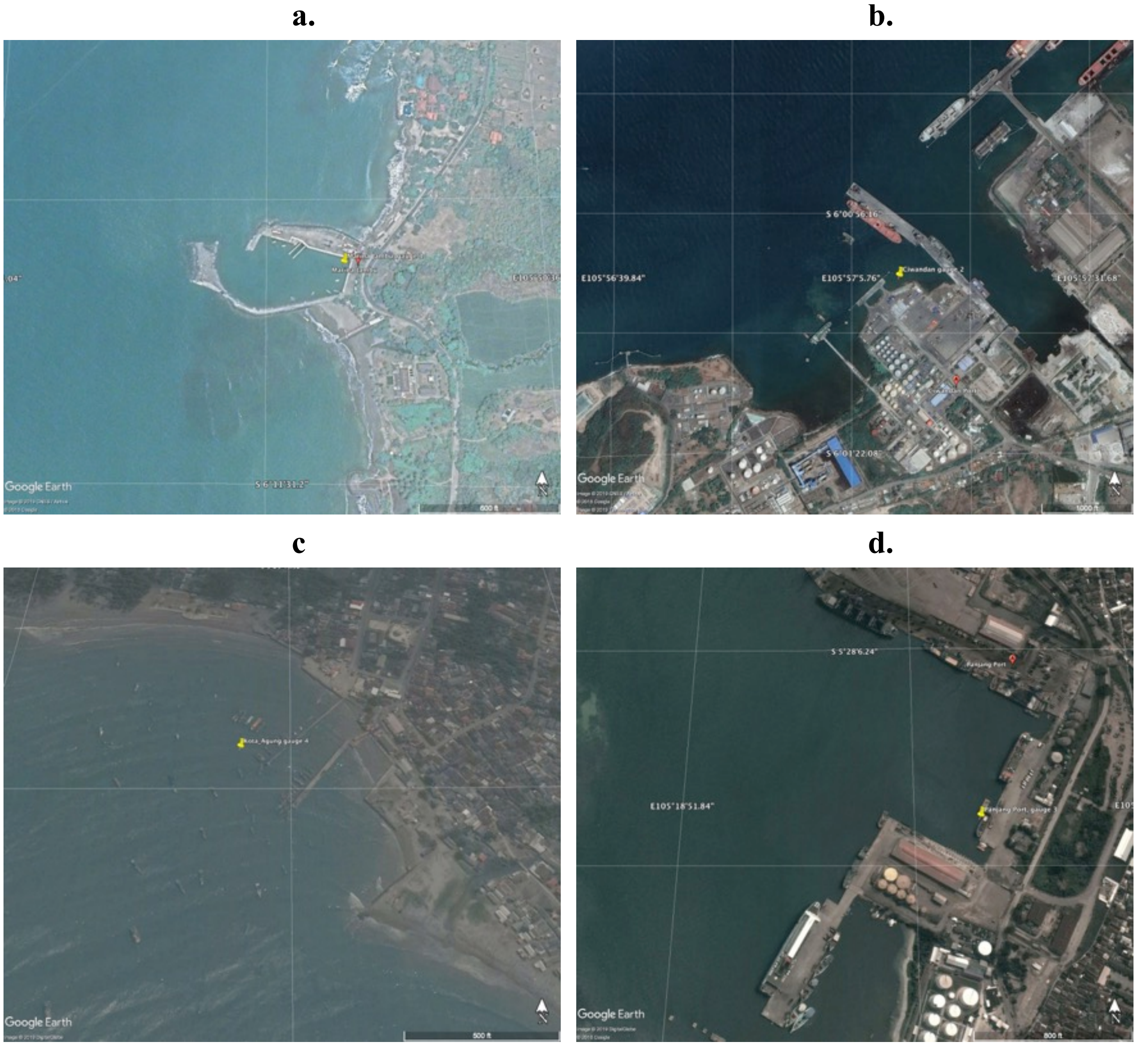

Figure S.4. GOOGLE EARTH images of the location of tide gauges 1-4 (Table 1, Fig. 1a): **a.** Marina Jambu, Serang; **b.** Ciwandan; **c.** Kota-Angung; **d.** Panjang.

Each of the tide gauges 1-4 used in this study (Ina-COAP, 2019; Fig. 1a; Table 1) is located near or within harbours/marinas or close to/behind a coastal structure (Fig S.4). Tide gauge 1, for instance, where the largest waves were recorded (Fig. S.4a), is located inside and at the far end of Marina Jambu (Fig. S.4a), which is about 200 by 100 m and not modelled in Grid 1 (especially at a 100 m resolution), within which the incoming tsunami would have triggered seiching oscillations. Assuming a quarter wavelength resonance within the shallow marina, an average depth of 3 m, the resonance period would be 2.5 min. Comparing the modelled and observed time series at gauge 1, additional sustained higher frequency oscillations (and hence less decay) can be seen in observations, particularly later in the measured data, than in the simulated data.

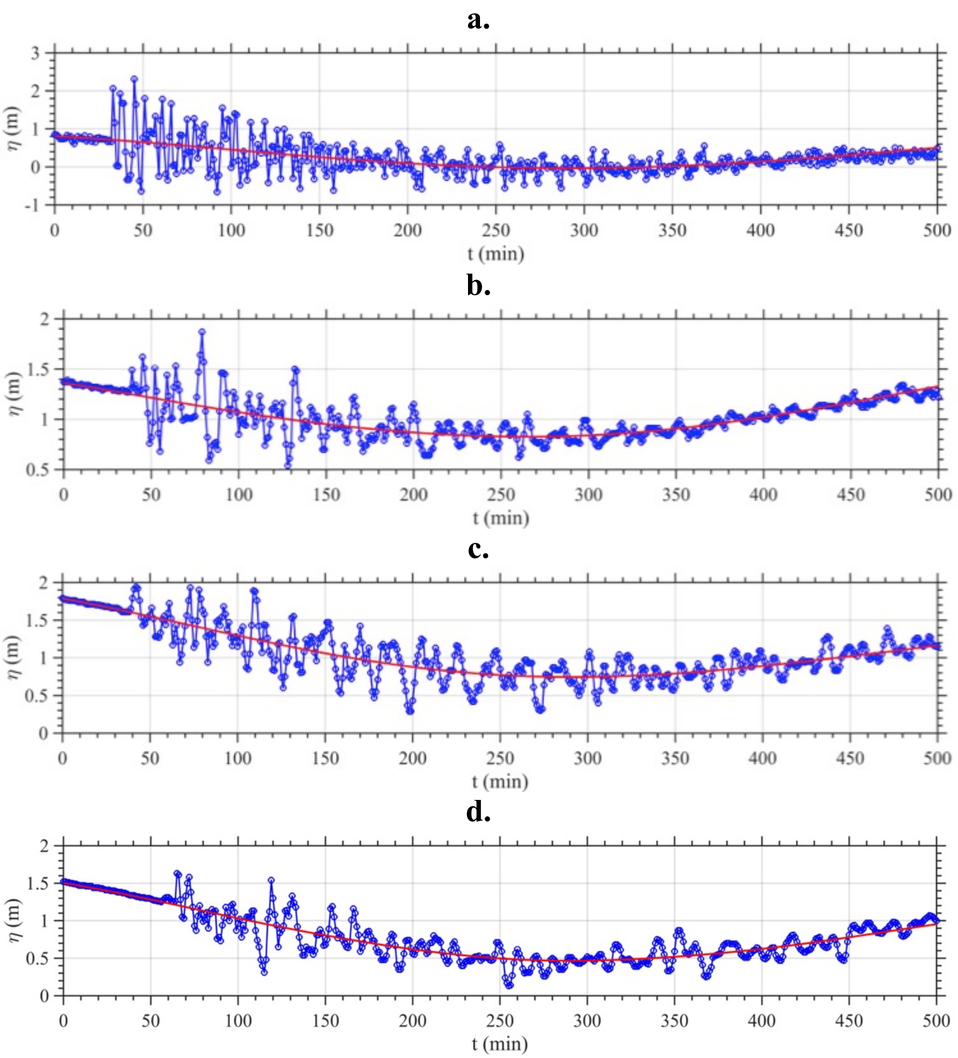

Figure S.5. Time series of surface elevations recorded at tide gauges 1-4 (Ina-COAP, 2019; Table 1, Figs. 1a, S.4) at 1 min intervals, using one of the functioning sensors (blue symbols). The red line shows the data filtered using a Butterworth filter (0.025, 0.00694 normalized passband/stopband frequencies, and 0.04 and 40 dB of passband/stopband attenuation). Time t = 0 is the start of the event estimated at 20:57’ local time (UTC + 7h) on December 22, 2018.

The raw surface elevation data, recorded at each tide gauge at 1 min intervals using 2 functioning sensors (Ina-COAP, 2019), was detided using a Butterworth filter and the Matlab *filtfilt* function. Based on the sampling period of 1 minute for the gauge data, the Butterworth filter parameters were set assuming 20 min and 12 h tsunami and tide periods, respectively, passband and stopband normalized frequency cutoffs of 0.025 and 0.00694, and 0.04 and 40 dB of passband and stopband attenuation, respectively. Note, the normalize frequencies vary in [0, 1] rad/sample. The raw and filtered data are shown in Fig. S.5. The measured tsunami elevations shown in Fig. 5 were obtained by subtracting the filtered data from the raw data. Although simulations were limited to about 130 min (Fig. 5), Fig. S.5 shows that significant waves were recorded for a much longer time at most tide gauges, due to multiple wave reflections and re-reflections in the Sunda Straits.

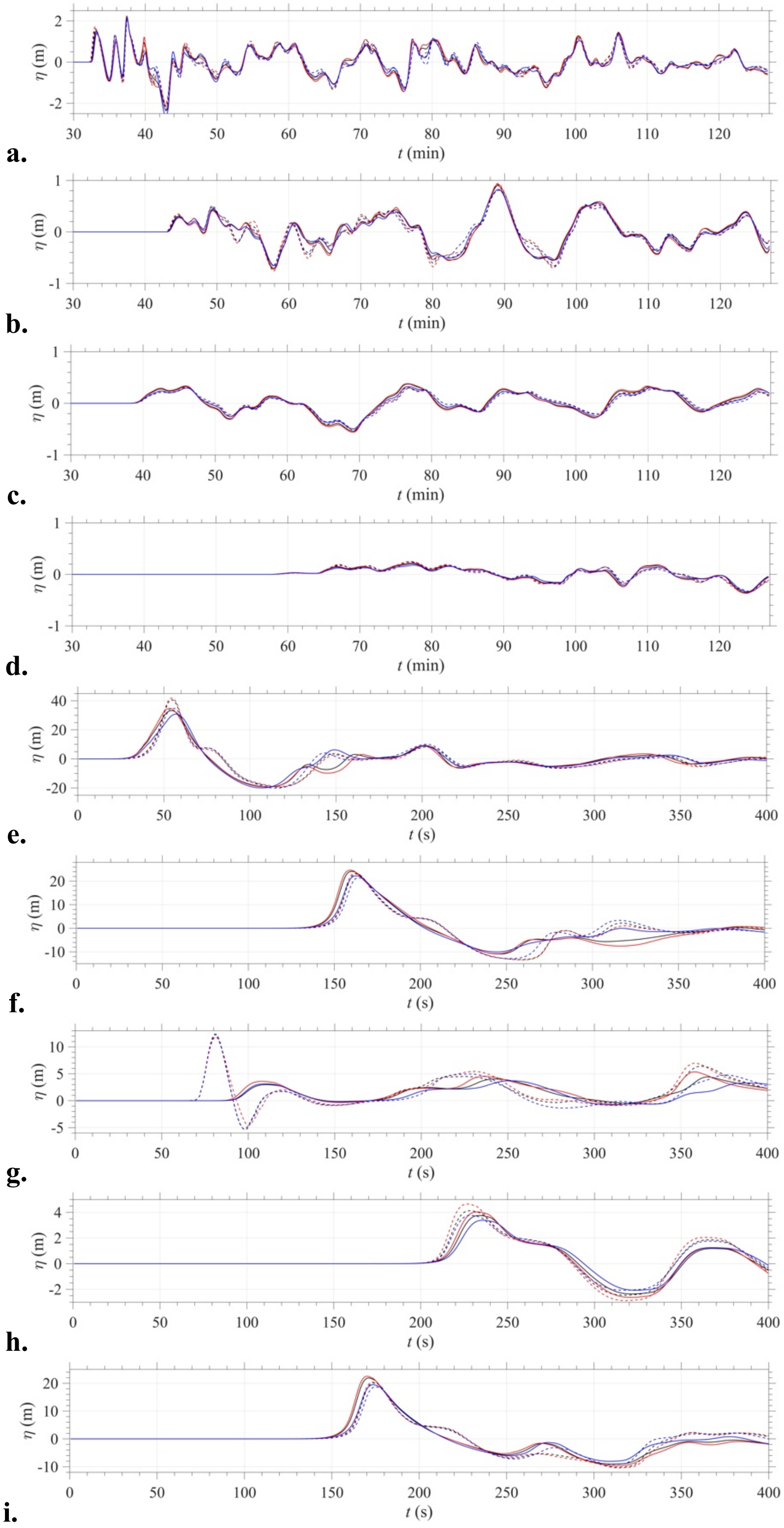

Figure S.6. Comparison of surface elevation time series computed at: (**a.-d.**) tide gauges 1-4 (Fig. 1a; Table 1) and (**e.-i.**) numerical wave gauges 5-9 (Fig. 1b; Table 1), for viscous (solid lines) and granular (dashed lines) collapse simulations: (black) likeliest 0.27 km^3, (red) 0.30 km^3 (+10%), and (blue) 0.22 km^3 (-20%) volumes.

Fig. S.6 illustrates the sensitivity of surface elevation time series computed at tide gauges 1-4 with FUNWAVE-TVD and numerical wave gauges 5-9 with NHWAVE (Figs. 1a,b; Table 1) for each of the tested collapse rheology (viscous or granular) and for 3 different collapse volumes, the likeliest 0.27 km^3, a larger 0.30 km^3 (+10%), and a smaller 0.22 km^3 (-20%) volumes (see Fig. 3). While larger differences between various cases occur in the near-field, at wave gauges located near AK, only small differences can be seen between the 6 cases in the far-field, at tide gauges, particularly early in the time series.

References

Ina-COAP. *Tide Gauge data in Indonesia*, <http://tides.big.go.id/las/UI.vm Accessed Jan. 3, 2019> (2019).

Supplementary File #4 – Simulated near-field tsunami impact vs. field data

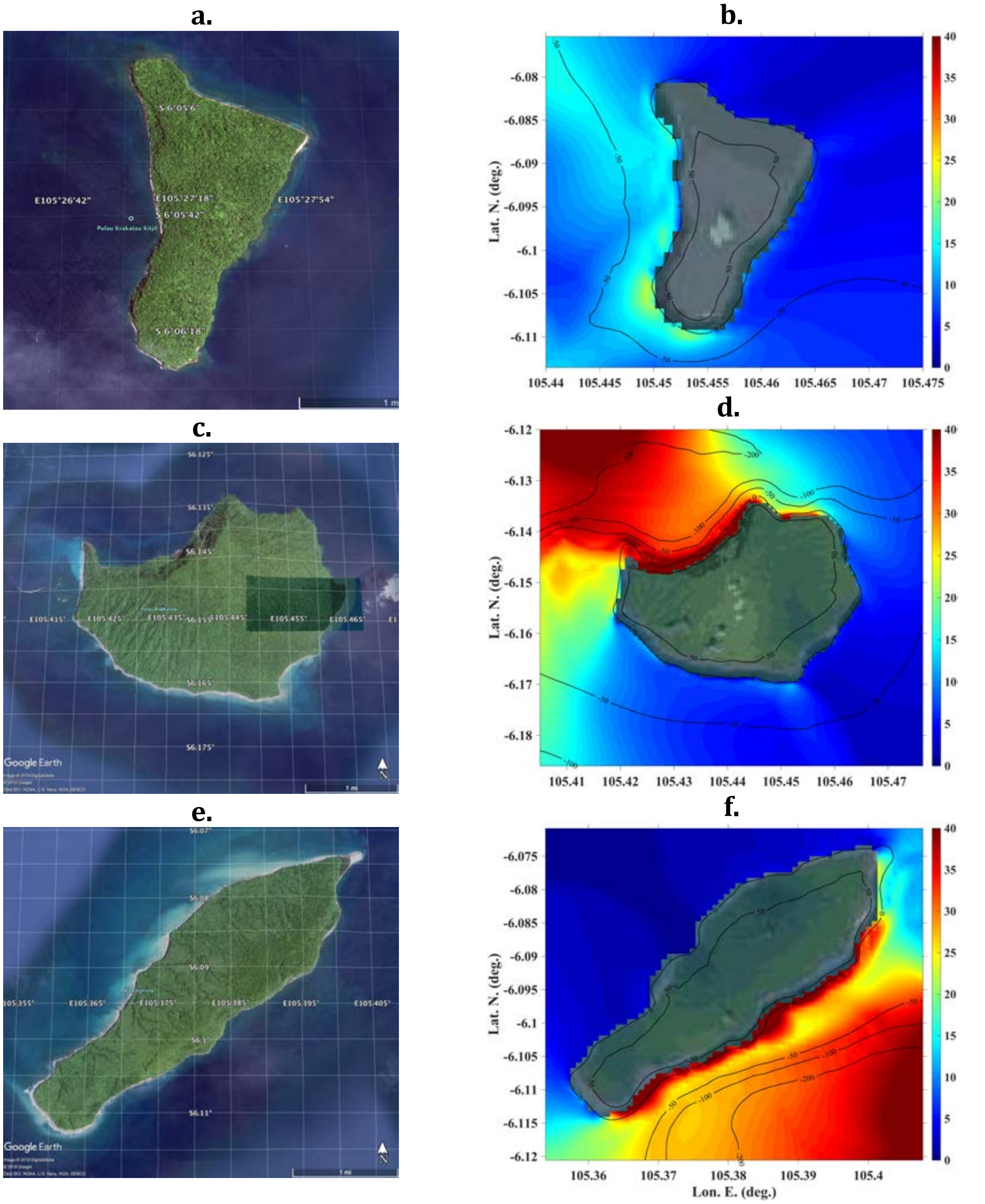

Figure S.7. (**a.,c.,e.**) Pre-event GOOGLE EARTH images (map data: 2019 GOOGLE DigitalGlobe) of Pajang, Rakata and Sertung, respectively (see also Fig. 1c); (**b.,d.,f.**) Zoom-ins of Fig. 4b results of maximum surface elevation simulated for the 0.27 km^3 volume (granular slide) on Panjang, Rakata and Sertung, respectively (colour scale in meter). Reference level is MSL + 1.5 m. See Fig. S.7 for field data observed in the same area. Maps in **b.**, **d.**, **f.** were produced by the authors based on the bathymetry/topography dataset, using MATLAB version 2017b; topography from GOOGLE EARTH georeferenced satellite images was embedded in these maps using an API key: GOOGLE EARTH, 2019 DigitalGlobe.

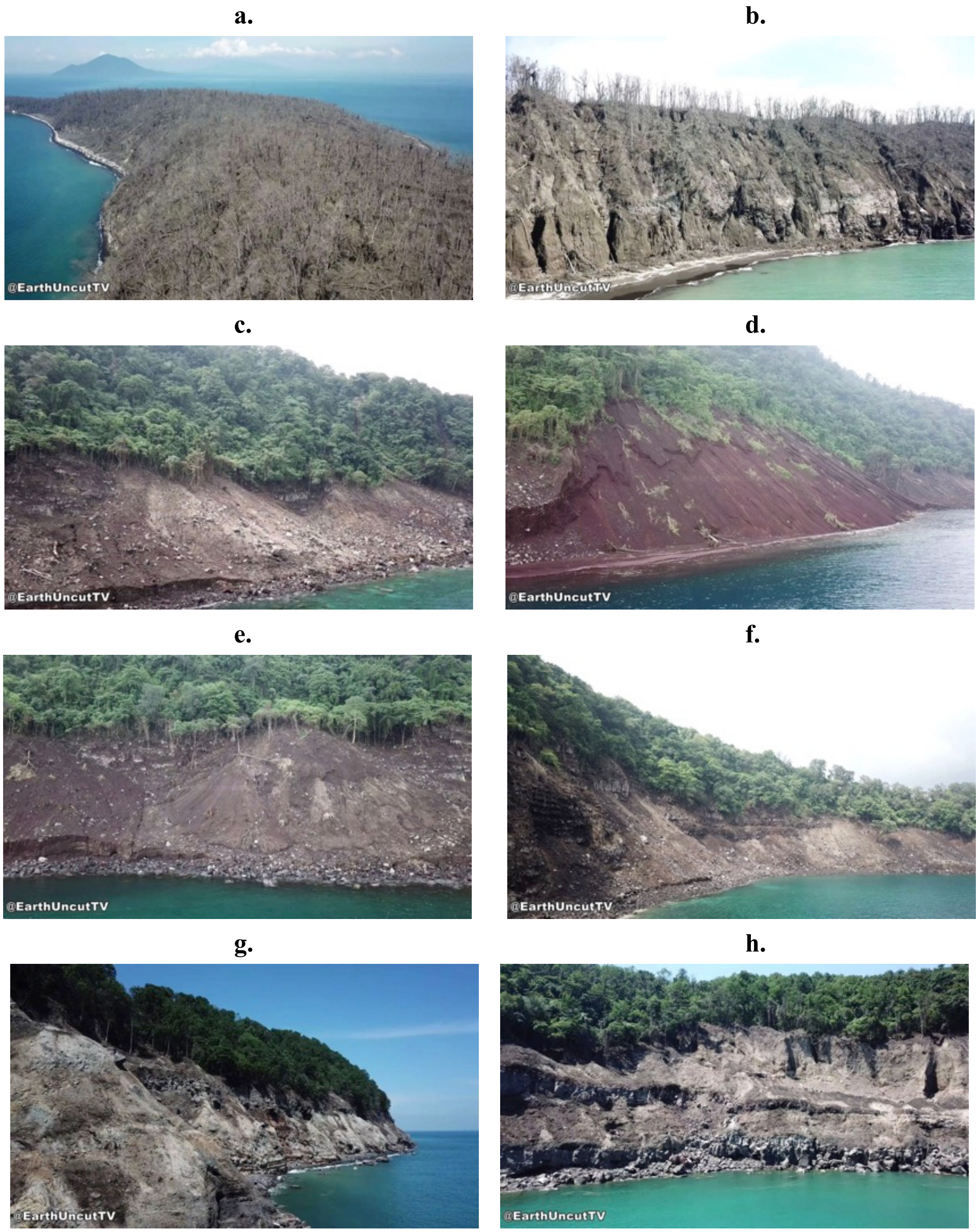

Figure S.8. Image sequence extracted from Reynolds' Jan.11, 2019 drone survey (reproduced with the author's permission) showing tsunami impact on (see Fig. 1 for locations): (**a.,b.**) Panjang's (Figs. S.7a,b) north and west coasts (Sebesi can be seen in the distance in (a), which positions the northern tip of Panjang); (**c.-f**). west coast of Rakata (Figs. S.7c,d); and (**g.,h.**) Southeast coast of Sertung (Figs. S.7e,f).

Figs. S.7a,c,e show pre-event GOOGLE EARTH images of Panjang, Rakata and Sertung, respectively, while Figs. S.7b,d,e show maximum surface elevations computed with

NHWAVE for the granular slide of 0.27 km^3 volume (zoom-in of Fig. 3b). Fig. S.8 shows images extracted from Reynolds' (2019) Jan. 11th, 2019 drone survey of the same area. Simulations results are shown in Fig. S.7 only for the 0.27 km^3 likeliest volume, with granular slide rheology, since results of all 6 cases simulated are qualitatively similar (and quantitatively close) results (see discussion in the main text).

In the simulations the north and northwest coasts of *Panjang* experienced 10-18 m runups, while the southwest and southern coasts experienced 10-23 m runups (Fig. S.7.b). These predictions appear to be supported by Figs. S.8a,b, although this will need to be confirmed by actual field surveys. In these figures, the northwest tip of the island shows signs of erosions and of some overtopping flow, which was strong enough to uproot most of the vegetation on the cliff-like coastline but not atop of it. There are also widespread signs of ash deposition. This is consistent with the more moderate tsunami generation on this side of AK (see animations in Suppl. #2).

In the simulations the northern coast of *Rakata* experienced 30 to nearly 50 m runups, while the western coast experienced 15-30 m runups (Fig. S.7.d). Figs. S.8c-e show images from the west coast of Rakata (likely form North to South), with clear signs of large runups, likely in the 20-50 m range, consistent with the predictions of Fig. S.7d; moreover, the image of Rakata in Fig. S.7c does not show any signs of significant pre-event erosion on this coast.

Finally, in the simulations, the eastern coast of *Sertung* experienced 20 (in the north) to nearly 50 m runups (Fig. S.7.f). Figs. S.8g,h show images from the southeast coast of Sertung (likely form South to North), with clear signs of large runups, likely in the 30-50 m range, consistent with the predictions of Fig. S.7f; here as well, the image of Sertung in Fig. S.7.e does not show any signs of significant pre-event erosion on this coast.

All of these observations will ned to be confirmed and quantified in actual field surveys, which the authors are planning to conduct in the near future.

References

Reynolds, J. *Post-collapse image of Anak Krakatau. Earth Uncut TV*, < Reynolds, J. *Post-collapse image of Anak Krakatau. Earth Uncut TV*, https://twitter.com/hashtag/Krakatau?src=hash, Accessed Jan. 11, 2019.> (2019).
